# Medical Convention of Ohio

**Published:** 1837

**Authors:** 


### Art. IV.—Medical Convention of Ohio.

Let not the physicians of Ohio forget that their second, or adjourned meeting, will be held at Columbus, on the first Monday of January next. The Convention of 1835, was attended by upwards of 70 members—that of 1838 will, no doubt, embrace twice the number. As far as we could judge, the members, generally, of the first Convention, were much gratified, and separated with the resolution to meet again—and not merely that, but to bring many of their friends with them.

A trip to our metropolis, in winter, is, in the present state of our roads, attended with some inconvenience; but, apart from the meetings and deliberations of the Convention, Columbus will afford much to compensate them. The General Assembly will be in session, and an opportunity afforded of seeing the Buckeye State perform her functions of legislation:—the school for the Deaf and Dumb, and that for the education of the Blind, will afford much matter for reflection, to those who study the physiology of the senses: lastly, the penitentiary will offer not a little, to interest all who desire to observe the effect on the constitution of man—physical, intellectual and moral—of a peculiar kind of regimen, enforced with unrelenting exactness.

But a higher reward for the sacrifices, which are required by such a trip, will be found in the deliberations of the Convention, on every subject connected with the advancement and elevation of the profession. And now is the time, for the whole of its members, to consider what should be presented for discussion. Even those who may be prevented from being there, may do some good, by communicating their views, to such of their friends as may be able to attend. The Convention of 1835, embraced in its periscope, a great variety of topics; but many of them were treated with brevity, and none of them were debated and discussed, to a degree commensurate with their importance. Not a few of them, therefore, may be reproduced at the next meeting, and subjected to a deeper analysis. Moreover, no doubt, a variety of new topics will be suggested; and many plans of future action and usefulness proposed and adopted.

But the highest enjoyment and the most substantial reward, derivable from the meeting, will consist in the opportunities for personal acquaintance, and pleasant interchange of feeling, that will be afforded. To gentlemen, who, from the nature of the practical duties of their profession, so frequently experience the reverse of this, those enjoyments will be peculiarly acceptable. They possess, however, a still higher value; for they contribute to purify and elevate the heart, and thus raise the character of the profession:--they tend to unite us into one brotherhood—to create an *esprit du corps*—to promote honorable emulation—to unitize our views and purposes, and to increase our moral power in the community.

The first idea of this Convention, originating, we believe, with Dr. Awl, of Columbus, has always seemed to us a happy thought—one from which important consequences are to flow, in a stream, which, we trust, will be permanent. Such results will not come, however, if we sit down and wait for the "moving of the waters." We must stir each other up. Those who live in remote situations, should be notified; recent emigrants should be informed; the newspaper press throughout the State should be brought into the service of those who can find time to write; and those who cannot, may obtain the publication, in the nearest paper, of this hasty article, the copy right of which has not been secured. D.

